# Unique tRNA Fragment Upregulation with SARS-CoV-2 but Not with SARS-CoV Infection

**DOI:** 10.3390/ijms25010399

**Published:** 2023-12-28

**Authors:** Isabella Imirowicz, Azeem Saifee, Leanne Henry, Leo Tunkle, Alexander Popescu, Philip Huang, Jibiana Jakpor, Ava Barbano, Rohit Goru, Audrey Gunawan, Maria Sicilia, Mori Ono, Xiaoyong Bao, Inhan Lee

**Affiliations:** 1 Outreach Division, miRcore, Ann Arbor, MI 48104, USA; 2 miRcore Volunteer Program, miRcore, Ann Arbor, MI 40104, USA; 3 Department of Pediatrics, The University of Texas Medical Branch, Galveston, TX 77555, USA; xibao@utmb.edu

**Keywords:** COVID-19, NRP1, SEMA3C, tRF5, small ncRNA, SARS-CoV, SARS-CoV-2, SARS, neural function, long COVID

## Abstract

Unlike other coronaviruses, severe acute respiratory syndrome coronavirus 2 (SARS-CoV-2) has rapidly infected the global population, with some suffering long-term effects. Thanks to extensive data on SARS-CoV-2 made available through global, multi-level collaborative research, investigators are getting closer to understanding the mechanisms of SARS-CoV-2 infection. Here, using publicly available total and small RNAseq data of Calu3 cell lines, we conducted a comparative analysis of the changes in tRNA fragments (tRFs; regulatory small noncoding RNAs) in the context of severe acute respiratory syndrome coronavirus (SARS-CoV) and SARS-CoV-2 infections. We found extensive upregulation of multiple tRFs in SARS-CoV-2 infection that was not present in SARS-CoV or other virus infections our group has studied. By comparing the total RNA changes in matching samples, we identified significant downregulation of TRDMT1 (tRNA methyltransferase), only in SARS-CoV-2 infection, a potential upstream event. We further found enriched neural functions among downregulated genes with SARS-CoV-2 infection. Interestingly, theoretically predicted targets of the upregulated tRFs without considering mRNA expression data are also enriched in neural functions such as axon guidance. Based on a combination of expression data and theoretical calculations, we propose potential targets for tRFs. For example, among the mRNAs downregulated with SARS-CoV-2 infection (but not with SARS-CoV infection), SEMA3C is a theoretically calculated target of multiple upregulated tRFs and a ligand of NRP1, a SARS-CoV-2 receptor. Our analysis suggests that tRFs contribute to distinct neurological features seen in SARS-CoV-2.

## 1. Introduction

Coronaviruses have been responsible for several notable viral outbreaks in the 21st century, including the severe acute respiratory syndrome (SARS) epidemic between 2002 and 2004, caused by SARS-CoV (CoV), and the coronavirus disease 2019 (COVID-19) pandemic, caused by SARS-CoV-2 (CoV2), which has persisted over 3 years and is ongoing. These two viruses, which share 79% of their genome sequences [1,2], primarily affect the respiratory system and are transmitted via droplets and particles emitted from an infected person’s nose or mouth. Spike proteins of both viruses bind the ACE2 receptor to enter human cells. Besides ACE2, the most prominent receptor, other receptors that mediate CoV2 infection of human cells include neuropilin-1 (NRP1), Eph receptors, transmembrane serine protease 2 (TMPRSS2), P2X7, and CD147 (BSG) [3,4].

Both CoV and CoV2 are neuroinvasive viruses able to directly enter brain tissue via olfactory bulb neurons, the blood-brain barrier, and other peripheral neural pathways. Neurological symptoms associated with CoV infection include epilepsy, muscle weakness, and chronic symptoms such as persistent fatigue, diffuse myalgia, depression, and sleep cycle disruption [5]. CoV2 infection has been linked to acute neurological symptoms including stroke, encephalopathies, anosmia, and ageusia [6]. Direct viral infection of neurons or glial and endothelial cells can lead to neuronal cell death while systemic infection can induce inflammation, vascular damage, and autoimmune responses that damage neural tissues [7].

Though both viruses can enter neural cells, the nature and timing of COVID-19 and SARS symptoms differ. While the neurological symptoms of SARS are mostly observed in the late disease stage, COVID-19 presents unique neurological symptoms such as anosmia during early CoV2 infection, even preceding respiratory symptoms [6]. Thus, CoV2 probably has additional ways of effectively interacting with neural cells, including olfactory receptor cells. Among CoV2 receptors, NRP1 is abundantly expressed in the respiratory and olfactory epithelium and is upregulated with CoV2 infection [8]. Blocking interactions between the CoV2 spike protein and NRP1 reduces viral infection [9].

Meanwhile, though sequencing technology advances have helped identify many types of human non-coding RNAs, functional studies are still in their infancy with regard to viral infection. Among the small non-coding RNAs, tRNA-derived fragments (tRFs) have specific functions related to external stresses such as viral infection or toxins. For example, overexpressed 5′-ends of tRNA-Gln-CTG fragments (tRF5-Gln-CTG) enhance respiratory syncytial virus replication [10]. Moreover, our group reported that certain tRF5s are significantly increased in COVID-19 patient nasal samples [11].

In this study, we utilized publicly available RNAseq data of cell lines infected by either CoV or CoV2 to identify differences in their mechanisms of infection. We found tRF5 expressions to be distinctively upregulated in CoV2-infected cell lines and nervous system-related KEGG pathways to be enriched among downregulated genes specific to CoV2. When we computationally predicted potential targets of these upregulated tRF5s with CoV2 infection without considering mRNA expressions, our posited targets showed enriched neurological functions. Considering the theoretical targets and genes only downregulated with COV2 infection, we propose an NRP1-CoV2 model which may account for early-onset neurological symptoms involving CoV2 infection with tRF upregulation. We thus propose measuring tRFs in long COVID patients to study long-term neurological symptoms.

## 2. Results

### 2.1. Uniquely Upregulated tRF5s with CoV2 Infection

**Figure 1:**
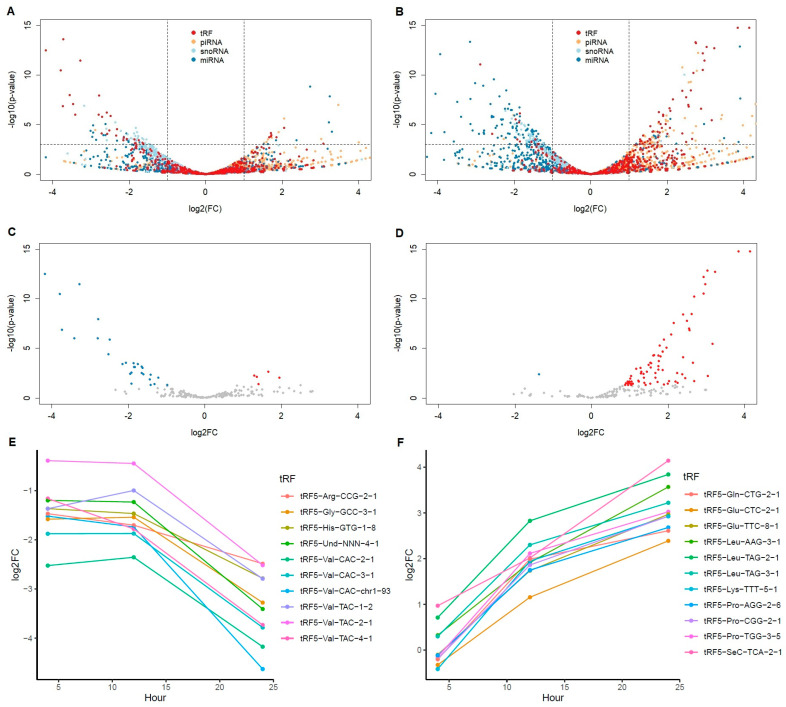
Differentially expressed tRF5s after SARS virus infection. (**A**,**B**) Volcano plots of all small RNAs with tRF (including all types of tRFs, red), piRNA (orange), snoRNA (light blue), miRNA (blue) at 24 h of SARS-CoV (**A**) and SARS-CoV-2 (**B**) infection in comparison to mock 4 h. Horizontal line is at *p* = 0.001. Vertical lines are at log2FC = ±1. (**C**,**D**) Volcano plots of only tRF5 species with read count more than 5 at any samples at 24 h of SARS-CoV (**C**) and SARS-CoV-2 (**D**) infection in comparison to mock 4 h. Colored dots are *p* < 0.05; red indicates upregulation (log2FC > 1) and blue downregulation (log2FC < −1). (**E**,**F**) Log2FC changes in comparison to mock 4h over time for the top 10 most significant tRFs for SARS-CoV (**E**) and SARS-CoV-2 (**F**) infection at 24 h. SARS-CoV-2 has one more tRF5, Glu-CTC (11th rank), included due to its importance and abundance.

To determine the molecular functions of CoV2 infection, we chose well-controlled Calu-3 (human epithelial cells from lung tissue) cell line data for our analysis. The GSE148729 dataset contains various samples of gene expression profile data, including human cell lines infected with CoV and CoV2 [12]. Since both small and total RNAseq data from the same cell lines were obtained for three different infection time points, we found these data useful in identifying regulatory small RNA functions in CoV2 infection. Though drawn from only two samples per condition, the time series data exhibits a clear trend. To reduce other variables, we used Calu-3 cell lines after 4 h of mock infection as a control with additional validation using 24 h of mock data only for the 24 h infection cases. We first analyzed small RNAseq data to understand expression changes after CoV2 infection. Similar to our previous report using patient nasal data [11], here in cell line experimental data we again found that tRFs, particularly tRF5s, were most distinctively upregulated with CoV2 among all small RNAs. Figure 1B shows a volcano plot focusing only on tRF5s after 24 h infection with CoV2, clearly demonstrating the upregulation of many tRF5s not seen with CoV infection (Figure 1A). Figure 1C,D shows the fold changes at different time points for tRF5s, with the top 10 most differentially expressed tRF5s (all upregulated and also found upregulated in patient nasal data) together with tRF5-Glu-CTC (11th, adjusted *p*-value < 10^−6^, upregulated and most abundant) at the 24 h infection time point, these changes increasing as the infection progressed. Among these upregulated tRF5s with CoV2 infection, tRF5-Gln-CTG and tRF5-Glu-CTC were selected for qRT-PCR validation and confirmed as upregulated in nasal samples of COVID-19 patients [11].

### 2.2. More Downregulated Genes with CoV2 Infection Than with CoV Infection

**Figure 2:**
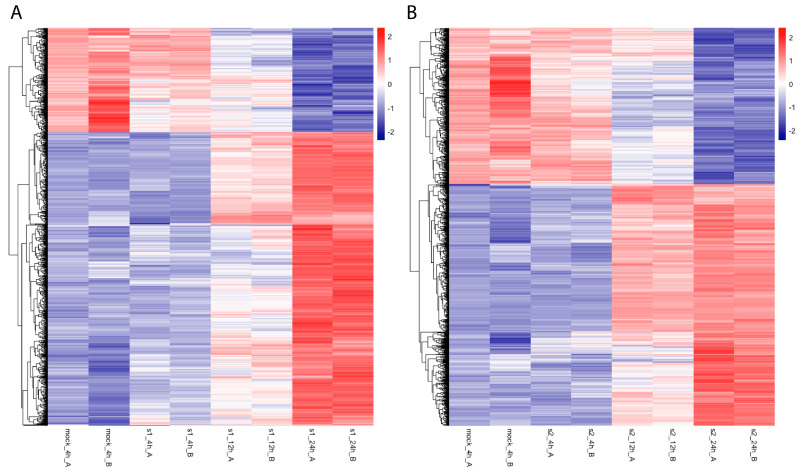
Heatmap of differentially expressed mRNAs (adjusted *p*-value < 10^−5^ at 24 h in comparison to mock 4 h) after SARS-CoV (**A**) and SARS-CoV-2 (**B**) infection. From left to right, mock 4 h and then infection time points at 4 h, 12 h, and 24 h of these gene expression levels are shown for each replicate A and B. After all SARS-CoV or SARS-CoV-2 infected samples were normalized using DESeq2, heatmaps were generated with normalized values of corresponding mRNAs using the R package heatmap with a scale-by-row option.

To investigate gene expression changes with CoV and CoV2 infection, we analyzed total RNAseq data and mapped them against gene-level expression values, combining all transcripts from the bowtie2 analysis. While more genes are upregulated than downregulated with both virus infections, CoV2 infection led to more genes being downregulated (Figure 2B) in comparison to CoV infection (Figure 2A). Such ratio differences (more downregulated genes in CoV2 than in CoV) decrease as the cutoff *p*-value increases, implying that the greater downregulation is specific to CoV2 infection. For example, with the adjusted *p*-value < 0.001 cutoff, 35.7% and 41.7% of differentially expressed genes were downregulated with CoV and CoV2 infection at 24 h, respectively (16.8% more downregulation in CoV2 infection). With the adjusted *p*-value < 0.05 cutoff, 45.2% and 48.6% of differentially expressed genes were downregulated with CoV and CoV2 infection at 24 h, respectively (7.5% more downregulation in CoV2 infection).

To shed light on the abundant tRF expressions with CoV2 infection, we compared expression patterns of genes potentially associated with tRF generation. Since tRNA is well known for its wide variety of modifications that respond to cellular environments [13] and tRNA stability is affected by methylation status, we checked tRNA methyltransferase and tRNA demethylase expression values for both virus infections. We also checked ANG, ELAC2, and DICER1, enzymes known to be associated with tRF biogenesis. For tRNA methyltransferases, we obtained the protein names from UniProt [14] and for tRNA demethylase, we checked *ALKBH1*, *ALKBH3*, and *FTO* gene expressions [15]. Among these, only differentially expressed genes with either CoV or CoV2 are shown in Table 1. Notably, while *DICER1* expressions were enhanced with both CoV and CoV2 infections, CoV2 infection increased *DICER1* expression 48% more than did CoV infection. Among all tRNA methyltransferases, *TRDMT1* (also known as *DNMT2*) was significantly downregulated only with CoV2 infection and not with CoV infection (Table 1).

To compare the functions of these downregulated genes using more comparable numbers to reduce statistical bias, we analyzed genes with an adjusted *p*-value < 0.05. Among the total 2292 and 2573 genes downregulated with CoV and CoV2 infection at 24 h, respectively, 1608 genes were commonly downregulated. Table 2 shows the enriched KEGG pathways for (1) commonly downregulated, (2) downregulated only in CoV infection, and (3) downregulated only in CoV2 infection at 24 h post-infection with adjusted *p* < 0.05. Commonly downregulated genes are involved in metabolism, neurodegenerative diseases, infectious diseases (bacterial), and translation processes. For CoV infection, proteasome (hsa03050) is the only pathway not found in the enriched pathways from commonly downregulated genes. On the other hand, CoV2 infection showed several specific enriched KEGG pathways, including the nervous system (hsa04723, hsa04728, hsa04730) and signal transduction (hsa04071, hsa04015), implying that additional molecular functions are disrupted with CoV2 infection.

### 2.3. Enriched Neural Functions of Theoretical Targets of Upregulated tRF5s

Given that many tRFs were uniquely upregulated and the ratio of the downregulated mRNAs increased with SARS-CoV-2 infection compared to SARS-CoV infection, we utilized tRF and mRNA expressions to identify tRF5 potential targets. We first performed theoretical hybridization calculations of uniquely upregulated tRF5s with entire untranslated regions (UTRs) of all coding transcripts.

Since most tRFs are longer than 30 nt, many mRNA UTRs have potential interaction sites for tRFs, making genome-wide analysis practically impossible. Furthermore, unlike the case for miRNA target prediction, there are minimal studies available for tRF target prediction. While tRF target research is limited, we here implemented two features in target prediction to reduce the number of potential theoretical interactions. First, we had previously identified that the 3′-end (not the 5′-end) of tRF5-Glu-CTC is functional in translational repression through targeting 3′ UTR [16]. Since we found that tRF5-Glu-CTC was also upregulated with SARS-CoV-2 infection, we used this feature as the first filter among all potential interactions between tRF5 and 3′ UTRs: 3′ UTR interacting with the 3′-ends of tRF5s. Second, among these tRF5-target pairs, we further filtered targets having simultaneous 5′ UTR interacting with the 5′-ends of tRF5s. In our earlier study, we observed a stronger translation repression when there were simultaneous interaction sites of miRNA with both the 5′ and 3′ UTR [17]. Since these sites are a subset of the tRF5s-3′ UTR interaction pairs, our theoretically predicted targets may have certain biology functions enriched, which would be meaningful in itself.

We then investigated the enriched functions of these theoretically predicted targets. To reduce redundant target gene lists from similar sequence tRF5s, we chose representative tRF5s as they were the most abundantly expressed among tRNAs translating the same amino acids. All enriched functions identified by String-db of the theoretical targets of these 6 representative tRF5s are shown in Table S1. All 6 tRF5 targets showed enriched gene ontology (GO) biological processes and 51 GO terms were found to be enriched in more than three tRF5 targets. Among these terms, we decided to focus on *neuro* or *nerv* terms, since others are general terms like metabolic, localization, signal, development, modification, and communication. Table 3 shows the enriched GO biological processes containing *neuro* or *nerv* terms among the enriched functions in Table S1. Targets of four tRF5s (tRF5-Glu-CTC-2-1, tRF5-Gln-CTG-2-1, tRF5-Leu-AAG-3-1, and tRF5-SeC-TCA-2-1) had enriched neural GO terms. To understand the significance of enriched GO biological processes, we randomly chose 1000 genes (average target number of the 6 tRFs = 995) among expressed mRNAs (total number 20,924) in Calu-3 cell lines and checked their enriched functions. Among the 10 random 1000-gene sets, five sets showed GO biology process terms enriched but none had neural functions.

Interestingly, the remaining two tRF targets are enriched in GO biological terms related to signal transduction. Among other enriched biological functions, targets of tRF5-Lys-TTT-5-1 are enriched in the GO component Synaptic vesicle (GO:0008021), and those of tRF5-Pro-TGG-3-5 are enriched in the KEGG Neurotrophin signaling pathway (hsa04772). These theoretical targets are general ones not confined to specific cell types.

We then determined which predicted tRF5 targets (having theoretical interaction sites) were also downregulated in Calu-3 cell lines with CoV2 infection but not with CoV infection. Among the target genes involved in Table 3 GO terms (thus focused on neural functions), the predicted targets from two or more of these 4 tRF5s which were also identified as downregulated only in CoV2 infection (adjusted *p* < 0.05) were ABAT, BCR, DHFR, DPYSL3, EEF2K, GLUD1, GRIN2A, HMGB1, MAPT, NDST1, NFIA, RIMS1, SEMA3C, STK25, and TACC1. While all other proteins are located mostly in the cell membrane or internal compartment, SEMA3C is mostly located in the extracellular compartment and is one of the ligands for NRP1 [18], which CoV2 uses as a receptor [8]. SEMA3C was predicted as a target of tRF5-Leu-AAG-3-1 and tRF5-SeC-TCA-2-1.

## 3. Discussion

Our cell line data analysis shows unique upregulation of the tRF5 family with CoV2 infection, not seen in other viral infection studies. Consistent with our nasal sample data from COVID-19 patients, tRF5-Gln-CTG and tRF5-Glu-CTC were also identified as upregulated in lung epithelial cell lines (Calu-3). Since cell line data have fewer variables, we identified additional upregulated tRF5s at 24 h after CoV2 infection. Most of these tRF5s only increased with CoV2 infection while additional mRNAs were downregulated in CoV2 infection compared with CoV infection, suggesting tRF5 functions merit close scrutiny with regard to COVID-19 research.

We greatly appreciate this kind of clearly defined paired sequencing data being publicly available so that citizen scientists can do small RNA research to uncover additional features with validation at each step. The unexpectedly highly upregulated tRF5s with CoV2 infection aligned well with expression value changes of genes currently known to be involved in tRF biogenesis. Only CoV2 significantly reduced expressions of *TRDMT1*, one of the well-known tRNA methyltransferases blocking tRF generation, while CoV2 increased expressions of *DICER1* more than did CoV, possibly helping tRF generation. Having confirmed the close alignment of mRNA data with tRF biogenesis, we further investigated mRNA expressions with regard to tRF functions to derive useful guidelines in future molecular functional studies of tRFs, as well as to provide insight into COVID-19 symptoms.

Since our study focus is tRF5s, we investigated downregulated mRNAs in more detail. General trends in mRNA changes due to both SARS virus infections were similar—cellular responses that help viral replication. Enriched KEGG pathways for commonly downregulated mRNAs in both CoV and CoV2 included multiple metabolism-related pathways. They also included functions to reduce various defenses such as lysosomes, peroxisomes, and thermogenesis. Bacterial invasion of epithelial cells GO-term was also enriched among these commonly downregulated genes, aligning well with the reduced co-infection among COVID-19 patients compared with those with influenza [19], presumably due to reduced competition with other pathogens during early infection. Enriched neurodegenerative disease pathways among commonly downregulated genes may be related to keeping cells alive for viral replication. CoV2 was reported to continuously replicate in Calu-3 cells even 72 h post-infection [20] and our analyzed data points were up to 24 h.

The enriched KEGG pathways of downregulated genes from CoV infection alone all fell under these same categories. However, enriched KEGG pathways of downregulated genes solely with CoV2 infection departed from these common features in ways such as including the nervous system and signal transduction, mostly involving neural cells. These changes were seen as early as 24 h post-infection of lung epithelial cells. While both CoV and CoV2 infections clearly affect neurological symptoms and we observed upregulated *NRP1* in both CoV and CoV2 infections, the key difference between them is the vast number of upregulated tRF5s. We propose that these upregulated tRF5s promote further enhancement of CoV2 interactions with neural cells.

In our earlier study, we detailed the interactions of tRF5-Glu-CTC with target gene *APOER2* and identified the 3′-end of tRF5-Glu-CTC as responsible for protein reduction through the *APOER2* 3′ UTR. Because only a few tRF5-target studies are available, we decided to expand tRF5-Glu-CTC interaction patterns with *APOER2* to all other tRF5 target predictions in this study. Additionally, we filtered the predicted targets to include additional interaction with the 5′ UTR of the same gene. Therefore, our predicted targets may miss other potential interactions. However, given the excess of possible targets for a given tRF5, we believe that our filters serve as a good starting point for tRF5 target analysis.

To our surprise, many target genes of upregulated tRF5 showed enriched functions related to the nervous system, features that were seen in downregulated mRNAs only with CoV2 infection. Since NRP1 has multiple functions in the immune system [21], *NRP1* expression may increase to enhance CoV2 replication. In addition, given our finding that the upregulation happens at an early infection time point, other mechanisms such as compensating for reduced neural functions may also be at play. When we focused on smaller groups of genes with neural functions, which are potential targets of multiple upregulated tRF5s and downregulated in infection with CoV2 but not with CoV, we were intrigued by SEMA3C. When we relaxed the distance criteria described in Methods from 19 nt to 16 nt, tRF5-Pro-AGG-2-6 also could target SEMA3C, which is expressed in most tissue types.

**Figure 3:**
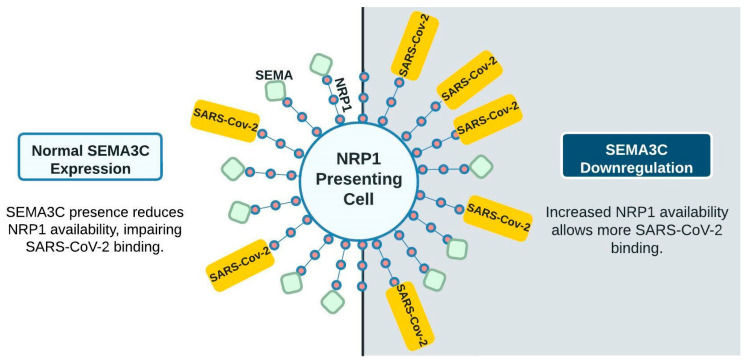
Proposed model of SARS-CoV-2 and SEMA competition for NRP1 interaction. With fewer ligands, more receptors are available for SARS-CoV-2 binding. Upregulated tRF5s are not shown in this diagram for clarity.

We therefore suggest a model involving tRF5-SEMA3C-NRP1 with CoV2 infection centered on neural functions. In Figure 3, the left panel represents the very initial CoV2 interaction, wherein most NRP1 receptors are occupied by other ligands such as SEMA3C (green squares), reducing the ability of CoV2 to bind to NRP1. The right panel shows CoV2 infection downregulating SEMA3C expression. With less SEMA3C, more NRP1 receptors are available for CoV2 binding, thus increasing the infection of other NRP1-presenting cells, including respiratory and olfactory epithelium. This is one example of highly upregulated tRFs possibly contributing to additional neurological symptoms not seen in other viral infections.

Some who recover from COVID-19 can experience long-term health effects (long COVID). Long COVID includes a variety of ongoing symptoms, lasting months or years and affecting different organs depending on the individual, and is thus not considered one illness. There are clearly multiple mechanisms leading to long COVID, including mitochondrial depletion [22]. Our findings point to a specific mechanism underlying neurological symptoms associated with long COVID. Recently, disability-adjusted life years (DALYs) were calculated based on 140,000 people who had COVID-19 compared with 6 million non-infected controls at the 2-year mark post-COVID. Neurologic DALYs per 1000 persons were found to be the most abundant, followed by cardiovascular cases [23]. While there is no current cure for long COVID, it would be interesting to see whether the tRF expressions are persistently enhanced in patients who have recovered from COVID-19.

Based on our paired data analysis, we suggest a model based on tRF5 upregulation specific to CoV2 infection and enriched neural functions both in mRNAs specifically downregulated in CoV2 and in theoretically predicted target mRNAs of the upregulated tRF5s. We have previously identified specific tRF5s upregulated in Alzheimer’s disease [24]. It seems that tRF5 functions are related to both infection and neurodegenerative diseases and may provide further insight into unique neurological symptoms such as anosmia with COVID-19.

## 4. Materials and Methods

### 4.1. Downloading Fastq Files from a GEO Dataset

We used gene expression omnibus (GEO) dataset GSE148729, which includes data from bulk and single-cell polyA RNA sequencing, small RNA sequencing, and total RNA sequencing of different human cell lines infected with CoV or CoV2 viruses. We selected Calu-3 (human epithelial cells from lung tissue) cell line samples of small RNA (GSM4477932 through GSM4477949) and total RNA sequencing data (GSM4477950 through GSM4477967). These samples included two replicates per condition at 4, 12, and 24 h, with appropriate controls at the 4 and 24 h time points. We used the fastq-dump command in the SRA toolkit [25,26] to download fastq files.

### 4.2. Aligning Sequencing to Transcripts

**Figure 4:**
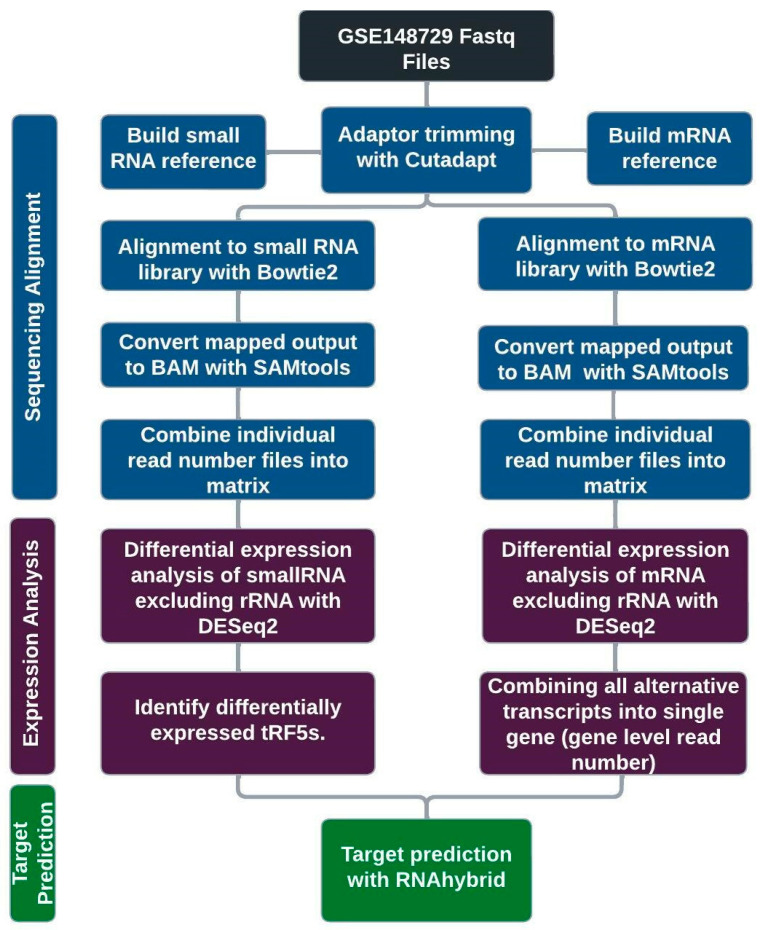
Schematics of sequencing data analysis. After sequence alignment, mRNA transcript read numbers were combined for gene-level analysis. Target predictions for upregulated tRF5s were calculated independently and then compared with their gene level expression changes.

We used cutadapt [27] to trim 3′-adaptor sequences and discarded read sequences of size less than 15. The sequences trimmed from the small RNAseq data were AAAAAAAAAA and TGGAATTCTCGGGTGCCAAGG, and the sequences trimmed from the total RNAseq data were AGATCGGAAGAGCACACGTCTGAACTCCAGTCA for pass 1 and AGATCGGAAGAGCGTCGTGTAGGGAAAGAGTGT for pass 2, as referenced from Illumina. We used bowtie2 [28] to align reads to reference sequences, allowing for one nucleotide mismatch for small RNAseq analysis. The reference for small RNAseq was built from combined small RNA sequences of (1) downloaded sequences of sno/miRNA tract using the UCSC Table Browser [29] and (2) prepared tRFs from 33 unique nucleotides of 5′-end of tRNAs (tRF5) and those of 3′-end (tRF3) as well as upstream pre-tRNA sequences (tRF1) of mature tRNAs. To build tRF references in more detail, we downloaded all tRNA identified from UCSC genome browsers and used the tRNA names as their root names. 33 nt is 1-4 nucleotides longer than most tRF5s and, based on years of observation, enough to effectively identify the tRFs using botwtie2 software (version 2.4.1). 53.5% of tRF5s were identified as redundant sequences and were thus removed, keeping only representative ones. For tRF3 references, we did not include ACC, the mature tRNA-added sequence, at the 3′-end. To build the reference for total RNA mapping, we downloaded NCBI RefSeq transcripts using the UCSC Table Browser. The mapped sequences were then processed using SAMtools [30] commands view, sort, index, and idxstats to obtain read counts for each transcript. Figure 4 shows the schematics of the process.

### 4.3. Differential Expression Analysis

We used DESeq2 [31] to identify differentially expressed small RNAs and mRNAs. Expression data at time points of 4, 12, and 24 h after infection were compared with the expression data of mock 4 h samples to keep the same reference point due to missing 12 h mock data. We separately used 24 h mock as a reference for the 24 h infection samples and compared the results to confirm that the results using the 4 h mock were valid (the top 12 most significantly upregulated tRF5s were the same except for one per each analysis; all were top-ranking upregulated tRF5s). Normalized expression values were also obtained using the DESeq2 process to generate heatmaps.

### 4.4. Involving Student Scientists

We invited high school students who had performed transcriptome analysis for over a year and reached conclusions on their research to join weekly RNAseq data analysis sessions with undergraduate mentors. After the students understood the research project, they divided the samples and performed specific steps of RNAseq data analysis each week, reporting their progress in a shared document. To ensure the quality of data analysis, students compared their results with those of counterparts during the weekly sessions after independently analyzing data. The results from the students’ individual analyses were reproduced using shell scripts for validation. All the confirmed results were used here and those who had processed data following all steps were invited to finalize this research as authors.

### 4.5. tRNA Methyltransferase Collection

We used UniProt [14] to identify tRNA methyltransferase. To specify the search terms, we used advanced search with function -> Enzyme classification [EC] value of Methyltransferases [2.1.1.-] and Keyword [KW] of tRNA* for human, yielding these 25 proteins: C14orf172, FTSJ1, H_YH95C04.1, LCMT2, METTL1, METTL2A, METTL2B, METTL6, METTL8, NSUN2, NSUN3, THUMPD3, TRDMT1, TRMO, TRMT1, TRMT10C, TRMT11, TRMT13, TRMT1L, TRMT2A, TRMT2B, TRMT44, TRMT5, TRMT61A, TRMT61B, TRMT9B, and TYW3.

### 4.6. Candidate Target Prediction

We downloaded 5′ and 3′ UTR of the NCBI RefSeq using the UCSC Table Browser. We then selected tRF5s differently expressed with SARS-CoV-2 infection and determined tRF5 sequences from the most abundant sequence reads in the bowtie2 analysis among the reads mapped to the initial 33-mer tRF5 database. We first used RNAhybrid [32] to identify all 5′ or 3′ UTR sites interacting with these tRF5 sequences with a hybridization energy of less than −25 kcal/mol. Next, we selected pairs with consecutive sequence matches of 8-mer or more. These tRF5-mRNA pairs were further narrowed down in two ways: (1) 3′-end sides of tRF5s interacting with 3′ UTRs [16] and (2) among these, 5′ UTR of the same mRNA interacting with the same tRF5 [17], but from the other side of the tRF5 and at least 19 nt away the 3′ UTR-interacting 8-mers. Note that our target prediction process yields a tRF5-target subset of conventional tRF5 interactions with 3′ UTR.

### 4.7. Enrichment Testing of tRF5 Targets

Since tRNAs carrying the same amino acid can have highly similar sequences, some tRF5s also have similar sequences, potentially leading to similar predicted targets. To avoid over-counting enriched functions among these potential targets, we chose representative tRF5s as they were the most abundantly expressed among tRNAs translating the same amino acids. We then analyzed enriched functions of these representative tRFs for the top 10 significant tRF5s using String-db [33].

## Figures and Tables

**Table 1:** Differentially expressed genes among methyltransferases, demethyltransferases, and ribonucleases at 24 h after infections.

Gene Symbol	SARS-CoV Infection		SARS-CoV-2 Infection	
	Log2FC	*p*-Value	Log2FC	*p*-Value
**tRNA Methyltransferase**				
*FTSJ1*	−0.41	0.058	−0.70	2.3 × 10^−4^
*METTL1*	−0.54	0.026	−0.17	0.53
*METTL8*	−0.53	0.052	−0.81	7.7 × 10^−4^
*THUMPD3*	0.24	0.17	0.36	0.022
*TRDMT1*	−0.30	0.41	−1.43	3.2 × 10^−5^
*TRMT2B*	−0.57	0.026	−0.37	0.13
*TRMT44*	0.59	0.036	0.67	0.60
*TRMT61A*	−0.56	0.0039	−0.15	0.49
*TRMT9B*	0.07	0.89	0.63	0.049
*TYW3*	−0.22	0.36	−0.42	0.033
**Demethylase**				
*ALKBH3*	−0.77	0.0022	−0.56	0.019
**Ribonuclease ^1^**				
*DICER1*	1.18	3.0 × 10^−29^	1.75	2.8 × 10^−69^

^1^ We checked only ANG, ELAC2, and DICER1, known to be involved in tRF biogenesis.

**Table 2:** Enriched KEGG pathways for downregulated genes (adjusted *p*-value < 0.05) after 24 h infection with SARS-CoV and SARS-CoV-2 compared to 4 h mock infection.

KEGG ID	KEGG Description	Observed Gene Count	Background Gene Count	False Discovery Rate
**Commonly downregulated genes**				
hsa03010	Ribosome	83	130	9.43 × 10^−36^
hsa01100	Metabolic pathways	209	1447	5.57× 10^−12^
hsa00190	Oxidative phosphorylation	45	130	1.83 × 10^−11^
hsa04714	Thermogenesis	61	229	1.83 × 10^−11^
hsa05012	Parkinson disease	57	240	3.47 × 10^−9^
hsa05014	Amyotrophic lateral sclerosis	68	352	9.68 × 10^−8^
hsa05010	Alzheimer disease	68	355	1.12 × 10^−7^
hsa05016	Huntington disease	60	298	1.75 × 10^−7^
hsa05020	Prion disease	52	265	3.40 × 10^−6^
hsa04142	Lysosome	29	126	0.00015
hsa04510	Focal adhesion	36	198	0.0011
hsa01212	Fatty acid metabolism	16	54	0.0015
hsa04932	Non-alcoholic fatty liver disease	29	148	0.0016
hsa03060	Protein export	10	23	0.0026
hsa04260	Cardiac muscle contraction	20	87	0.003
hsa00062	Fatty acid elongation	10	25	0.0039
hsa05110	Vibrio cholerae infection	13	48	0.0105
hsa05100	Bacterial invasion of epithelial cells	16	70	0.012
hsa01200	Carbon metabolism	22	117	0.013
hsa04810	Regulation of actin cytoskeleton	32	209	0.0219
hsa03008	Ribosome biogenesis in eukaryotes	16	77	0.0246
hsa05205	Proteoglycans in cancer	30	196	0.0279
hsa04146	Peroxisome	16	79	0.0282
hsa00010	Glycolysis/Gluconeogenesis	14	65	0.031
hsa04910	Insulin signaling pathway	22	133	0.0415
**Downregulated only in SARS-CoV**				
hsa03010	Ribosome	19	130	0.00021
hsa05012	Parkinson disease	26	240	0.00021
hsa05016	Huntington disease	30	298	0.00021
hsa05020	Prion disease	28	265	0.00021
hsa05014	Amyotrophic lateral sclerosis	30	352	0.0011
hsa01100	Metabolic pathways	81	1447	0.0015
hsa05010	Alzheimer disease	28	355	0.0051
hsa03050	Proteasome	8	43	0.0116
hsa00190	Oxidative phosphorylation	14	130	0.0134
hsa04932	Non-alcoholic fatty liver disease	15	148	0.0137
**Downregulated only in SARS-CoV-2**				
hsa01100	Metabolic pathways	149	1447	4.78 × 10^−14^
hsa00280	Valine, leucine, and isoleucine degradation	12	46	0.0021
hsa05132	Salmonella infection	27	209	0.0021
hsa00100	Steroid biosynthesis	8	20	0.0025
hsa00640	Propanoate metabolism	9	34	0.0094
hsa01200	Carbon metabolism	17	117	0.0099
hsa04723	Retrograde endocannabinoid signaling	19	145	0.012
hsa05031	Amphetamine addiction	12	66	0.012
hsa05110	Vibrio cholerae infection	10	48	0.0121
hsa04071	Sphingolipid signaling pathway	16	116	0.0153
hsa00020	Citrate cycle (TCA cycle)	7	29	0.036
hsa00190	Oxidative phosphorylation	16	130	0.036
hsa00340	Histidine metabolism	6	21	0.036
hsa00620	Pyruvate metabolism	8	38	0.036
hsa04728	Dopaminergic synapse	16	128	0.036
hsa04730	Long-term depression	10	59	0.036
hsa00564	Glycerophospholipid metabolism	13	97	0.0375
hsa04015	Rap1 signaling pathway	21	202	0.0375
hsa04962	Vasopressin-regulated water reabsorption	8	44	0.0478

**Table 3:** Enriched GO biological process terms including *neuro* or *nerv* for tRF5-predicted target genes.

Term ID	Term Description	tRF5 Name
GO:0001764	Neuron migration	tRF5-Glu-CTC-2-1
GO:0007399	Nervous system development	tRF5-Gln-CTG-2-1
		tRF5-Glu-CTC-2-1
		tRF5-Leu-AAG-3-1
GO:0007417	Central nervous system development	tRF5-Glu-CTC-2-1
		tRF5-Leu-AAG-3-1
GO:0010975	Regulation of neuron projection development	tRF5-SeC-TCA-2-1
GO:0010976	Positive regulation of neuron projection development	tRF5-Glu-CTC-2-1
GO:0022008	Neurogenesis	tRF5-Gln-CTG-2-1
		tRF5-Glu-CTC-2-1
GO:0030182	Neuron differentiation	tRF5-Gln-CTG-2-1
		tRF5-Glu-CTC-2-1
		tRF5-Leu-AAG-3-1
GO:0031175	Neuron projection development	tRF5-Leu-AAG-3-1
GO:0045664	Regulation of neuron differentiation	tRF5-Glu-CTC-2-1
		tRF5-SeC-TCA-2-1
GO:0045665	Negative regulation of neuron differentiation	tRF5-Glu-CTC-2-1
GO:0045666	Positive regulation of neuron differentiation	tRF5-Glu-CTC-2-1
GO:0048666	Neuron development	tRF5-Gln-CTG-2-1
		tRF5-Glu-CTC-2-1
		tRF5-Leu-AAG-3-1
GO:0048699	Generation of neurons	tRF5-Gln-CTG-2-1
		tRF5-Glu-CTC-2-1
		tRF5-Leu-AAG-3-1
GO:0048812	Neuron projection morphogenesis	tRF5-Leu-AAG-3-1
GO:0050767	Regulation of neurogenesis	tRF5-Glu-CTC-2-1
		tRF5-Leu-AAG-3-1
GO:0050769	Positive regulation of neurogenesis	tRF5-Glu-CTC-2-1
GO:0051960	Regulation of nervous system development	tRF5-Gln-CTG-2-1
		tRF5-Glu-CTC-2-1
		tRF5-Leu-AAG-3-1
GO:0051962	Positive regulation of nervous system development	tRF5-Glu-CTC-2-1

## Data Availability

The data presented in this study are available in supplementary materials.

## Supplementary Materials

The following supporting information can be downloaded at: https://www.mdpi.com/article/10.3390/ijms25010399/s1.
